# Consequences of child emotional abuse, emotional neglect and exposure to intimate partner violence for eating disorders: a systematic critical review

**DOI:** 10.1186/s40359-017-0202-3

**Published:** 2017-09-22

**Authors:** Melissa Kimber, Jill R. McTavish, Jennifer Couturier, Alison Boven, Sana Gill, Gina Dimitropoulos, Harriet L. MacMillan

**Affiliations:** 1 Department of Psychiatry and Behavioural Neurosciences, McMaster University, 1280 Main Street West, Hamilton, ON L8S 4K1 Canada; 2 Offord Centre for Child Studies, McMaster University, 1280 Main Street West, MIP Suite 201A, Hamilton, ON L8S 4K1 Canada; 3 Department of Health Research Methods, Evidence, and Impact, McMaster University, 1280 Main Street West, Hamilton, ON L8S 4K1 Canada; 4 Department of Pediatrics, McMaster University, 1280 Main Street West, Hamilton, ON L8S 4K1 Canada; 5 Faculty of Social Work, University of Calgary, 2500 University Dr. NW, Calgary, AB T2N 1N4 Canada

**Keywords:** Child maltreatment, Emotional abuse, Emotional neglect, Child exposure to intimate partner violence, Eating disorders

## Abstract

**Background:**

Child maltreatment and eating disorders are significant public health problems. Yet, to date, research has focused on the role of child physical and sexual abuse in eating-related pathology. This is despite the fact that globally, exposure to emotional abuse, emotional neglect and intimate partner violence are the three of the most common forms of child maltreatment. The objective of the present study is to systematically identify and critically review the literature examining the association between child emotional abuse (EA), emotional neglect (EN), and exposure to intimate partner violence (IPV) and adult eating-disordered behavior and eating disorders.

**Methods:**

A systematic search was conducted of five electronic databases: Medline, Embase, PsycINFO, CINAHL, and ERIC up to October 2015 to identify original research studies that investigated the association between EA, EN and children’s exposure to IPV, with adult eating disorders or eating-disordered behavior using a quantitative research design. Database searches were complemented with forward and backward citation chaining. Studies were critically appraised using the Quality in Prognosis Studies (QUIPS) tool.

**Results:**

A total of 5556 publications were screened for this review resulting in twenty-three articles included in the present synthesis. These studies focused predominantly on EA and EN, with a minority examining the role of child exposure to IPV in adult eating-related pathology. Prevalence of EA and EN ranged from 21.0% to 66.0%, respectively. No prevalence information was provided in relation to child exposure to IPV. Samples included predominantly White women. The methodological quality of the available literature is generally low. Currently, the available literature precludes the possibility of determining the extent to which EA, EN or child exposure to IPV have independent explanatory influence in adult eating-related pathology above what has been identified for physical and sexual abuse.

**Conclusions:**

While a large proportion of adults with eating disorders or eating-disordered behavior report EA, EN, or child exposure to IPV , there is a paucity of high-quality evidence about these relationships.

**Electronic supplementary material:**

The online version of this article (10.1186/s40359-017-0202-3) contains supplementary material, which is available to authorized users.

## Background

Eating disorders, including anorexia nervosa (AN), bulimia nervosa (BN), binge eating disorder (BED), as well as other specified feeding or eating disorder (OS-FED, previously eating disorder not otherwise specified (ED-NOS)), are serious psychiatric conditions characterized by a significant and persistent shift in eating and weight-related behavior. Recent population-based surveys of adults in the United States indicate that the life time prevalence of these disorders is as follows: 0.6% (AN), 1.0% (BN), 2.8% (BED), and 4.6% (ED-NOS/OS-FED) respectively [1, 2]. Tending to have onset in the adolescent period, eating disorders are chronic conditions and those who experience longstanding eating-disordered behavior, are more likely to experience recurring inpatient hospital admissions; the rate of hospitalization due to EDs and the length of stay has increased by 40% or greater for children and adolescents in Canada and the US since the early 2000’s [3, 4].

Child maltreatment, which includes physical, sexual and emotional abuse (EA), physical and emotional neglect (EN) and child exposure to intimate partner violence (IPV) [5]—is increasingly being recognized as a non-specific risk factor for EDs and eating-disordered behavior. As a public health concern in its own right, child maltreatment experiences are also associated with significant increases in one’s risk for mood and anxiety disorders, substance use disorders, and alcohol use disorders [6, 7], all of which have been found to co-occur at high rates among adolescents and adults with eating and weight-related pathology [8, 9]. Thus far, the literature investigating the relationship between child maltreatment and EDs has tended to focus on physical and sexual abuse [10], with much less attention on the potential influence of child exposure to IPV, EA and EN on disordered-eating onset and duration. This is a critical research gap given that globally child EA, EN and exposure to IPV constitute three of the most prevalent forms of child maltreatment. For example, recent meta-analyses report a global self-reported lifetime prevalence of 36% and 18.4% for EA and EN respectively [11]. Child exposure to IPV – which includes child exposure to the intentional use of physical, sexual, or verbal violence between their adult caregivers – ranges from 10 to 20%, depending on child or adult retrospective self-reports [12].

Work by Caslini and colleagues [10] offers some important insights regarding possible differential relationships between EA, EN, and eating-related pathology, as well as the current state of the evidence in this field. For example, the authors identified a significant and positive association between childhood EA, BN and BED, calling into question the disproportionate focus on physical and sexual abuse as risk factors for eating disorders [10]. With respect to anorexia, the authors found no significant association between this form of eating disorder and childhood exposure to EA. In addition, significant heterogeneity was identified across the included studies, suggesting that pooling the results from the studies estimating the relationship between child EA and anorexia nervosa is not appropriate.

Of note, methodological, conceptual and substantive decisions informing the Caslini et al. [10] review complicate the generalizations that can be made from these findings. For example, child EA was considered “an act of omission and commission, which is judged based on a combination of community standards and professional expertise to be psychologically damaging. It is committed by parents or significant others who are in a position of differential power that render the child vulnerable, damaging immediately or ultimately the behavioral, cognitive, affective, social and physiological functioning of the child” ([10], p. 80). However, evidence from the child maltreatment field indicates that EA (acts of commission) and EN (acts of omission) are distinct forms of child abuse with physiological and psychological consequences [13]. In addition, emerging literature suggests that EN may have a specific relationship to different forms of eating-disordered pathology which are distinct from the impacts of EA; EN may be more strongly associated with bingeing behaviors and EA more strongly associated with binge-purge cycles (e.g. [14]). These emerging findings warrant an independent synthesis of the literature evaluating the empirical relationships between EA, EN and eating disorders.

Importantly, previous literature has suggested that symptoms of child and adolescent EDs are associated with significant distress among caregivers, which may place caregivers at increased risk for perpetrating emotionally abusive or emotionally neglectful behaviors towards their ill child [15, 16]. These findings indicate that the relationship between EA, EN, and eating-disordered behaviors may be inversely related, or even, reciprocal. Similarly, the last two decades have seen an emergence of work evaluating the extent to which child maltreatment may indirectly influence the onset of eating-disordered behavior through various social and psychological processes that can confer greater susceptibility to the development of eating-related pathology. Two examples include the role of depressive symptoms and emotion dysregulation. A recent paper by Michopoulos et al. [17] indicates that depressive symptoms and emotion dysregulation fully mediated the association between childhood EA and eating-disordered behavior (e.g. eating when lonely, eating less to avoid weight gain, eating when depressed, etc.) among a population-based sample of low income, inner-city adults. Unfortunately, the scope of Caslini and colleagues ' work [10] did not allow for the consideration of these conceptual and substantive concerns in their synthesis, nor did their review include the potential role of child exposure to IPV in the onset and duration of eating-disordered experiences.

There is also a great deal of uncertainty concerning the prevalence and characteristics of child maltreatment and eating disorders across the population more generally. Both are considered to be vastly underreported to health and social service professionals [18, 19], which is further complicated by a systemic and cultural under-acknowledgement of eating-disordered behavior (e.g. excessive exercise, dieting, fasting, etc.) and extreme weight-loss as physiologically and psychologically damaging [20]. In addition, there is considerable stigma associated with both child maltreatment and eating disorders, such that many individuals living with these experiences will not come to the attention of health and social service professionals [21, 22]. Thus, given that sub-clinical eating-disordered behavior is predictive of clinical eating-disorder onset [23], a synthesis of the literature which considers the evidence by which EA, EN, and child exposure to IPV are associated with eating-disordered behavior, as well as clinically diagnosed eating disorders is important in understanding the relationship between these forms of child maltreatment and eating-disorder pathology. Given that evidence indicates that health and social service professionals experience significant challenges in identifying EA, EN, and child exposure to IPV, and that these can be the most difficult forms of child maltreatment to identify, assess and respond to [13, 24, 25], a comprehensive and critical synthesis of the adult literature presents an opportunity to attune practitioners, researchers, and advocates to the intersection of these experiences, facilitate greater awareness to their co-occurrence in the adult population, and to leverage the need for appropriate responses to these experiences within prevention and intervention contexts.

The current paper utilizes systematic search and critical review methodology [26] to synthesize quantitative studies evaluating the relationship between child exposure to IPV, EA, EN and adult eating disorders and eating-disordered behavior. Secondary objectives are: (a.) to identify the most commonly used measures of child exposure IPV, EA, EN, eating disorders and eating-disordered behavior within quantitative studies; (c.) to describe the theoretical models, if any, informing investigations of the relationship between these forms of family violence and eating-disordered behavior; (d.) to identify the extent to which studies evaluate the intersection of these experiences across important sub-groups of the population (e.g. ethnic minorities, immigrants, males); and (e.) to characterize the existing knowledge gaps within this area of research.

## Methods

### Identification of literature

The systematic search (unregistered) was conducted by an information scientist (JRM) with significant experience in literature searches related to family violence and health outcomes. Index terms and keywords related to childhood exposure to EA, EN, or IPV (e.g., “intimate partner violence,” “domestic violence,” “battering,” “child abuse,” “maltreatment,” “abuse”), eating disorders (e.g. “eating disorders,” “mental disorders,” “bulimia,” “anorexia,” “eating disorder not otherwise specified”) and eating-disordered behavior (e.g. “laxative,” “purging,” “diet,” “vomiting”) were used and were generated, reviewed and approved by the research team (see Additional File 1 for Medline search strategy). As per standard search procedures, definitional variability of key concepts, constructs, or terms can be captured through the purposeful and strategic utilization of index terms and proximity operators [27]. In this regard, our search implemented the use of index terms (e.g., “mental disorders/”, “child abuse/”) in all databases to help ensure that definitional variations for our primary constructs (e.g. emotional abuse) were captured. Similarly, keywords were combined by proximity operators and were selected based on the test of a sample of articles that were eligible for inclusion in the review [27]. These strategies and corresponding searches were run in the following databases from database inception (indicated in brackets) to October 26, 2015: Medline (1946-), Embase (1947-), PsycINFO (1806-), CINAHL (1981-), and ERIC (1966-). The titles and abstracts of all articles identified by our database searches were screened by at least one reviewer. One hundred titles and abstracts were independently screened by all reviewers involved in this stage of the screening process to ensure adequate agreement between reviewers (*n* = 3). Estimates of agreement between reviewers ranged from 0.6 to 0.8, demonstrating moderate to strong agreement in screening. At the level of title and abstract screening, an article suggested for inclusion by one reviewer was sufficient to put it forward to full-text review. Forward and backward citation chaining of the included articles was conducted during the week of September 19, 2016. This was done to complement the search and to locate any possible articles that: (a.) may have been published between the initial database search and the authoring of this manuscript and (b.) might have been missed by the initial database search. Additional database search strategies, as well as the audit trailing relating to citation chaining procedures, is available by request from the corresponding author.

### Study selection criteria

Inclusion criteria were as follows: (a.) primary studies with adult samples (≥ 18 years of age) that used a quantitative design; (b.) published articles; (c.) investigations which reported a numerical estimate of correlation or effect (that could be converted to a correlation coefficient) between respondents’ self-reported exposure (i.e. exposure prior to 18 years of age) to EA or child exposure to IPV, or EN and current eating disorder or eating-disordered behavior (self-reported or clinically diagnosed); and (d.) English-language articles only. Excluded studies include (a.) all non-quantitative designs; (b.) non-primary studies and non-journal articles (e.g. reviews, dissertations, master’s theses, book chapters); (c.) studies in which information about childhood experiences of EA, EN or exposure to IPV was based on child welfare records or samples recruited from child welfare or criminal justice organizations/settings; and (d.) studies which combined child maltreatment variables, such that data specific to the effect of EA, EN and exposure to IPV could not be extracted. Excluding dissertations and grey literature from the present review was a pragmatic decision and largely directed by the size of the returned database results. However, this decision is bolstered by recent evidence that suggests that the inclusion of grey literature, including dissertations, rarely alters the outcomes of quantitative syntheses [28].

### Data extraction

A standardized template for data extraction of key information was completed for each article. Information extracted included that which pertained to the publication characteristics (year of publication, full citation, country of data collection), design characteristics (longitudinal versus cross-sectional design, primary versus secondary data), sample characteristics [(total sample used in analysis, sampling frame (clinical, versus community, versus college sample), proportion of women, proportion of racial/ethnic minorities, proportion of immigrants)], prognostic and outcome measurement characteristics (type of child maltreatment investigated, type of eating disorder or eating-disordered behavior investigated, specific prognostic and outcome measure used), mediators and moderator s evaluated (if relevant), inclusion of a theoretical model, consideration of socio-economic disadvantage, as well as relevant effect estimate information on the association between EA, EN, exposure to IPV and the eating-disorder outcomes). With this information in mind, it is important to note that our search strategy was conceptualized and implemented so as to identify the quantitative literature investigating the association between the child maltreatment variables of interest and adult eating-related pathology. Our extraction strategy, however, focused on identifying, collating and synthesizing information pertinent to the article characteristics described above. Notably, extraction of theoretical models took the form of identifying whether or not the authors explicitly stated that their study, research objectives, and/or analytical approach was informed by any previously published theoretical framework. In this regard, the name of the framework/model was extracted and the original authors of the framework/model was extracted, as was a description of the framework/model. Two reviewers (MK, AB) independently extracted the data, which was cross-verified.

### Quality appraisal

The Quality in Prognosis Studies (QUIPS) tool was used to assess risk of bias across six domains: study participation, study attrition, prognostic factor measurement, outcome measurement, study confounding and statistical analysis and reporting [29]. One reviewer (MK) independently completed the appraisal tool for each study and classified the level of bias for each domain. An overall classification of study bias (i.e. low, moderate, or high risk of bias) was assigned to each article following the processes and recommendations made by the tool authors [29]. The methodological quality of a given study was classified with low bias if the study was determined to have low bias across each of the six methodological domains; moderate bias if they received a ‘low bias’ assignment on four or five of the six QUIPS domains; and high bias if they had three or less domains classified as low bias. Classifications for each study on each domain of the QUIPS tool as well as the overall classification of study bias were independently confirmed by a second reviewer (SG); discrepancies in classification were resolved through consensus discussions between reviewers. Only three discrepancies on domain classification were identified, which were then resolved through discussion.

## Results

**Fig. 1:**
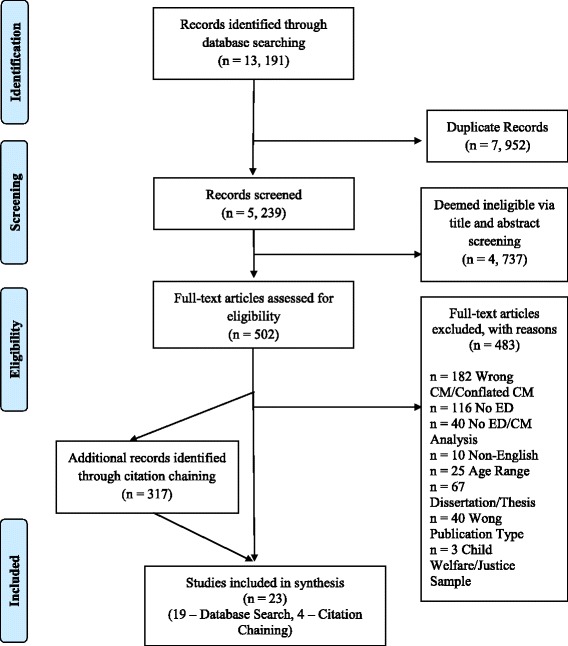
PRIMSA Flow Diagram. Legend: The PRISMA diagram details the search and selection process applied during our systematic literature search and critical review

A total of 13,191 records were identified and, after deduplication, 5239 title and abstracts were screened using the above criteria (see Fig. 1). After full-text screening of 502 articles, 19 articles were included in this review. An additional 317 articles were identified by the forward and backward citation chaining procedures and were then screened in their full-text form. Four additional articles were identified through citation chaining procedures for inclusion in this review, resulting in a total of 23 articles.

### Study characteristics and methodological quality

Overall, the methodological quality of the included studies was low. Only one of the 23 included studies received a classification of low study bias [30] (See Table 1). The remaining 22 articles received a classification of high bias. With respect to specific classifications on the QUIPS tool, a large proportion of the included studies were classified with high or moderate bias on the domains of: study participation, study attrition, prognostic factor measurement and study confounding. Alternatively, 48% (*n* = 11) of the studies received a low bias classification in the domain of statistical analysis and reporting, and 52% (*n* = 12) received the same classification in relation to the domain of outcome measurement.

**Table 1:** Overall Classification of Study Bias for Each of the Included Sources

Overall Bias Classification	Study ID (Reference)
Low Bias	Mason et al. [30]
Moderate Bias	________
High Bias	Feinson and Hornik-Lurie [32], Utzinger et al. [33], Afifi et al. [52], Michopoulos et al. [17], Moulton et al. [14], Brooke and Mussap [41], Burns et al. [40], Becker and Grilo [36], Bardone-Cone et al. [49], Gentile et al. [42], Messman-Moore and Garrigus [43], Wonderlich et al. [44], Fosse and Holen [45], Kugu et al. [39], Grilo and Masheb [37], Schoemaker et al. [34], Grilo and Masheb [38], Witkiewitz and Dodge-Reyome [46], Kent et al. [47], Mullen et al. [51], Rorty et al. [50], van der Kolk et al. [31]

The included studies represent the experiences of 38,161 participants. Sixteen of the 23 studies focused exclusively on women and four additional studies reported that women constituted 80% or greater of their sample respondents. None of the included sources explicitly focused on males. Ten of the sources were unclear with respect to the proportion of their sample that identified as a racial or ethnic-minority. Among those that did report this information (*n* = 14), the proportion of racial and ethnic minorities in a given sample ranged from 1% to 97.4%. Similarly, a large proportion of the included studies (*n* = 21, 91.3%) did not report the immigrant status of their sample participants. Half of the included studies did not explicitly report their country of data collection. Of those that did, most studies took place in high-income countries (one each in Canada, Norway, New Zealand, the Netherlands, four from the United States, two from the United Kingdom) and one study took place in the middle-income country of Turkey. Finally, over 80% of the included studies were published since the year 2000, with 37.5% of the studies published within the last five years. Publication of the articles included in this synthesis span nearly two-and-a-half decades, with the earliest published in December of 1991 [31] and the most recent published (online-first) in May of 2016 [32].

### Prevalence of child maltreatment among adults with eating disorders and eating-disordered behavior

Among those studies focusing on clinically diagnosed eating disorders [*n* = 9; 33, 34, 37, 42–45, 48, 49], five reported a prevalence rate for the type(s) of child maltreatment investigated. Prevalence estimates for EA among participants with BN came from three studies and ranged from 27.8% to 43.8% [33–35]. Three studies provided prevalence estimates for EA among participants with BED, with the rate ranging from 24.1% to 53.0% [36–38]. The work by Kugu [39] indicated that 38.1% of the participants who met clinical criteria for an eating disorder in their study (*n* = 21, 18 BN, 3 BED) reported experiencing EA in childhood.

Information about the prevalence of childhood EN among those with BN was only available from one study; Schoemaker and colleagues [34] indicated that among their sample of 38 women who met clinical criteria for BN, 47% reported EN in childhood. Among participants with clinically diagnosed BED, the prevalence of EN in childhood was reported by three studies and ranged from 21.1% to 66.0%, respectively [36–38]. Importantly, there is no available information pertaining to the prevalence of childhood exposure to IPV among adults with any form of eating disorder and none of the studies provided prevalence estimates pertaining to EA or EN among adults living with AN, avoidant restrictive food intake disorder (ARFID), or OS-FED.

Among the 15 studies which explore the association between our child maltreatment variables and adult eating-disordered behavior [14, 17, 30–32, 36, 38, 40–47], only one provided child maltreatment prevalence estimates. In the work by Mason and colleagues [40], prevalence of binge eating among participants who reported a slight, moderate or significant childhood history of EA differed and were reported as 31.8%, 41.3% and 52.5% respectively.

### Relationship between EA, eating-disordered behavior and eating disorders

Tables 1 through 3 provide details concerning each of the included studies and classify each of the included sources according to the strength of the bivariate association reported by (or computed for) each of the respective papers for the child maltreatment variable of interest (i.e. EA, EN or child exposure to IPV), eating disorders and eating-disordered behavior.

Most studies (19 of 23, Table 2) focused on child EA and eating-related pathology rather than EN (Table 3) or exposure to IPV (Table 4). Seven of these 19 studies considered the influence of childhood EA on the onset or prevalence of eating disorders, as determined by structured diagnostic interviews. An additional eleven studies considered the influence of this form of child maltreatment on self-reported eating-disordered behavior and one source considered the role of child EA on both eating disorders and eating-disordered behavior [38]. The strength of association between child exposure to EA and a clinically diagnosed eating disorder ranged from weak (0.16; [48]) to exceptionally strong (0.89; [33]); estimates of the association between this form of maltreatment and eating-disordered behavior ranged from very weak (0.03; [41]) to moderately strong (0.47; [30]). Importantly, sample sizes for the respective studies ranged from 41 to 4377 participants and 89.5% of these sources (*n* = 17) utilized a cross-sectional design.

**Table 2:** Sources focusing on childhood exposure to emotional abuse

Author, Country	Design (cross-sectional, longitudinal)	Sample Type (college, community, primary care, tertiary psychiatric care)	Sample Size (n)	Sample Characteristics • % Female • % Ethnic-minority • % Immigrant	Age Range (years)	Strength of Correlation for EA
Eating Disorders						
Utzinger et al. [33], USA^*^	cross-sectional	tertiary psychiatric care, college, community	*n* = 133	• 100% • 3% • Unclear	18 to 55	> 0.85^a**^
Bardone-Cone et al. [49], USA^*^	cross-sectional	community, tertiary psychiatric care	*n* = 138	• 100% • 13% • Unclear	18 to 55	0.11–0.25^a**^
Kugu et al. [39] Turkey	cross-sectional	college	*n* = 42	• 85.7% • Unclear • Unclear	18 to 24	0.51–0.75^c**^
Schoemaker et al. [34], Netherlands	cross-sectional	community	*n* = 1926	• 100% • Unclear • Unclear	18 to 45	0.26–0.50^a**^
Grilo and Masheb [37], USA*	cross-sectional	primary care, tertiary psychiatric care	n = 1, 241	• 97.9% • 19.8% • Unclear	18 to 65	0.26–0.50^c^
Mullen et al. [51], New Zealand	cross-sectional	community	*n* = 497	• 100% • Unclear • Unclear	18 and over	0.26–0.50^a**^
Rorty et al. [50], USA*	cross-sectional	community, college, tertiary psychiatric care	*n* = 120	• 100% • Unclear • Unclear	18 to 35	0.11–0.25^a**^
Eating-Disordered Behavior						
Feinson and Hornik-Lurie [32], Jerusalem	cross-sectional	primary care	*n* = 498	• 100% • Unclear • Unclear	21 and older	0.11–0.25^a**^
Mason et al. [30], USA	longitudinal	community	*n* = 4, 377	• 100% • ~ 3.2% • Unclear	22 to 29	0.11–0.25^ad**^ 0.26–0.50^ae**^
Michopoulos et al. [17], USA	cross-sectional	primary care	n = 1, 110	• 80.4% • 97.4% • Unclear	18 to 65	0.11–0.25^c**^
Moulton et al. [14], Scotland	cross-sectional	college	*n* = 142	• 100% • Unclear • Unclear	18 to 46	0.26–0.50^a**^
Brooke and Mussap [41],	cross-sectional	college	*n* = 299	• 52% • Unclear • Unclear	18 to 40	0.01–0.10^cf^ 0.11–0.25^b**^ 0.26–0.50^a**^
Burns et al. [40], USA*	cross-sectional	college	n = 1, 254	• 100% • 22.3% • Unclear	18 to 22	0.01–0.10^a^ 0.11–0.25^a**^
Becker and Grilo [36], USA^*^	cross-sectional	tertiary psychiatric care	*n* = 137	• 100% • 15% • Unclear	20 to 59	0.01–0.10^af^
Messman-Moore and Garrigus [43], USA*	cross-sectional	college	*n* = 289	• 100% • 9% • Unclear	18 to 22	0.26–0.50^a**^
Wonderlich et al. [44], USA*	longitudinal	college, community, tertiary psychiatric care	*n* = 123	• 100% • 3.3% • Unclear	18 to 55	0.11–0.25^a**^
Witkiewitz and Dodge-Reyome [46], USA	cross-sectional	college	*n* = 88	• 100% • 1.0% • Unclear	18 to 25	0.26–0.50^a**^
Kent et al. [47], United Kingdom	cross-sectional	college	*n* = 236	• 100% • Unclear • Unclear	18 to 48	0.11–0.25^a**^ 0.26–0.50^a**^
Eating Disorders and Eating-Disordered Behavior						
Grilo and Masheb [38], USA*	cross-sectional	primary care, tertiary psychiatric care	n = 1, 270	• 97.3% • 19.8% • Unclear	18 to 65	0.11–0.25^c**^

^*^ Country of data collection not articulated. Assumption of country location was made given language used to describe participants (e.g. African American) or based upon identification of the location of the study’s Institutional Review Board

^**^ Authors reported at least one bivariate correlation estimate to be significant at *p* < .05

^a^Estimate falls within this range among women

^b^ Estimate falls within this range among men

^c^Estimate falls within this range among men and women

^d^ Estimate computed through converting risk ratios to odds ratios, and then, to a correlation coefficient. Correlation represents strength of correlation between moderate abuse exposure prior to the age 11 years and lifetime binge eating disorder after age 11

^e^ Estimate computed through converting risk ratios to odds ratios, and then, to a correlation coefficient. Correlation represents strength of correlation between severe abuse exposure prior to the age 11 years and binge eating disorder after age 11

^f^ Estimate reported was non-significant

**Table 3:** Sources focusing on childhood exposure to emotional neglect

Author, Country	Design (cross-sectional, longitudinal)	Sample Type (college, community, primary care, tertiary psychiatric care)	Sample Size (n)	Sample Characteristics • % Women • % Ethnic-minority • % Immigrant	Age Range (years)	Strength of Correlation for EN
Eating Disorders						
Utzinger et al. [33], USA^*^	cross-sectional	tertiary psychiatric care, college, community	n = 133	• 100% • 3% • Unclear	18 to 55	0.76–0.85^a**^
Bardone-Cone et al. [49], USA^*^	cross-sectional	Community, tertiary psychiatric care	*n* = 138	• 100% • 13% • Unclear	18 to 55	0.11–0.25^a**^
Grilo and Masheb [37], USA*	cross-sectional	primary care, tertiary psychiatric care	*n* = 1, 241	• 97.9% • 19.8% • Unclear	18 to 65	0.26–0.50^c**^
Schoemaker et al. [34], Netherlands	cross-sectional	community	n = 1926	• 100% • Unclear • Unclear	18 to 45	0.26–0.50^a**^
Eating-Disordered Behavior						
Michopoulos et al. [17], USA	cross-sectional	primary care	n = 1, 110	• 80.4% • 97.4% • Unclear	18 to 65	0.11–0.25^c**^
Moulton et al. [14] Scotland	cross-sectional	college	n = 142	• 100% • Unclear • Unclear	18 to 46	0.26–0.50^a**^
Brooke and Mussap [41],	cross-sectional	college	n = 299	• 52% • Unclear • Unclear	18 to 40	0.01–0.10^b**^ 0.11–0.25^c**^ 0.26–0.50^a**^
Becker and Grilo [36], USA^*^	cross-sectional	tertiary psychiatric care	*n* = 137	• 100% • 15% • Unclear	20 to 59	0.01–0.10^ad^
Fosse and Holen [45], Norway	cross-sectional	tertiary psychiatric care	*n* = 107	• 100% • Unclear • Unclear	18 to 55	0.11–0.25^ad^
Eating Disorders and Eating-Disordered Behavior						
Grilo and Masheb [38], USA*	cross-sectional	primary care, tertiary psychiatric care	n = 1, 270	• 97.3% • 19.8% • Unclear	18 to 65	0.01–0.10^cd^ 0.11–0.25^cd^

* Country of data collection not articulated. Assumption of country location was made given language used to describe participants (e.g. African American) or based upon identification of the location of the study’s Institutional Review Board

** Authors reported at least one bivariate correlation estimate to be significant at *p* < .05

^a^ Estimate falls within this range among women

^b^ Estimate falls within this range among men

^c^ Estimate falls within this range among men and women

^d^ Estimate reported was non-significant

**Table 4:** Sources focusing on childhood exposure to intimate partner violence

Author, Country	Design (cross-sectional, longitudinal)	Sample Type (college, community, primary care, tertiary psychiatric care)	Sample Size (n)	Sample Characteristics • % Women • % Ethnic-minority • % Immigrant	Age Range (years)	Strength of Correlation for child exposure to IPV
Eating Disorders						
Afifi et al. [52], Canada	cross-sectional	community	n = 23, 395	• Unclear • 16% • 18%^a^	18 and over	0.26–0.50^bd**^
Eating-Disordered Behavior						
Gentile et al. [42], USA*	cross-sectional	college	*n* = 884	• 56% • 80.7% • 71.7%	18 to 40	0.11–0.25^bf^
van der Kolk et al. [31], USA	longitudinal	Community, tertiary psychiatric care, primary health care	*n* = 74	• 52.7% • Unclear • Unclear	18 to 39	0.01–0.10^bcef^ 0.11–0.25^bef^

^*^ Country of data collection not articulated. Assumption of country location was made given language used to describe participants (e.g. African American) or based upon identification of the location of the study’s Institutional Review Board

^**^ Authors reported at least one bivariate correlation estimate to be significant at *p* < .05

^a^Proportion of respondents indicating they were born outside of Canada

^b^Estimate falls within this range among men and women

^c^Based on follow-up data for longitudinal study

^d^Estimate computed through converting odds ratio for AOR1in Afifi et al. (2014) to Pearson correlation coefficient

^e^ Based on baseline data for longitudinal study

^f^ Estimate reported was non-significant

Among the eight sources reporting on the relationship between EA and clinically diagnosed eating disorders, four focused on BN [33, 34, 49, 50], two focused on BED [37, 38], one combined diagnostic sub-types in their analyses (e.g. BN and BED; [39]), and one did not identify a specific eating disorder of interest [51]. None of the studies examined EA in relation to AN, ARFID, or OS-FED.

With respect to the eleven sources evaluating the influence of EA on self-reported eating-disordered behaviors, five sources examined more than one type of eating-disordered behavior [38, 40, 41, 43, 47]. Across the eleven sources, four considered bingeing [30, 32, 38, 40], one source considered purging [40], one considered eating restraint [38], two sources considered general bulimic symptomology [43, 47], one source considered emotional eating [17], three sources considered participant's drive for thinness [41, 43, 47], one source considered participant's drive for muscularity [41], and six sources evaluated participant' s generalized eating-disordered behavior [14, 36, 40, 44, 46, 47]. None of the included sources considered excessive exercise, laxative, diuretic or steroid use or abuse.

### Relationship between EN, eating-disordered behavior and eating disorders

The characteristics of the studies examining child EN in relation to adult eating-related pathology are included in Table 3. Four of the 23 sources considered the influence of childhood EN on the onset or prevalence of eating disorders, as determined by structured diagnostic interviews. An additional five sources considered the influence of EN on self-reported eating-disordered behavior. One source considered the role of EN in eating disorders as well as eating-disordered behavior [38]. The strength of the correlation between child exposure to EN and a clinically diagnosed eating disorder ranged from weak-to-moderate (0.21; [49]) to very strong (0.76; [33]), with the strength of correlation between this form of maltreatment and eating-disordered behavior ranging from very weak (0.03; [41]) to moderately strong (0.34; [14]). Sample sizes for these studies ranged from 107 to 1296 participants and all studies utilized a cross-sectional design.

Among the studies that examined the relationship between EN and clinically diagnosed eating disorders, three focused on BN [33, 34, 49], two on BED [37, 38], and one considered both BN and AN [45]. None of the studies examined EN in relation to neither ARFID nor OSFED.

With respect to the six sources evaluating the influence of EN on self-reported eating-disordered behaviors, three sources examined more than one type of eating-disordered behavior [38, 41, 45]. The following behaviors were examined in one study: bingeing [38], eating restraint [38], emotional eating [17], drive for thinness [41] and drive for muscularity [41]. Two sources evaluated participants’ generalized eating-disordered behavior [14, 36] and one source evaluated participants' self-report of bulimic and anorexic symptomology [45]. None of the sources considered purging, excessive exercise, laxative, diuretic or steroid use or abuse.

### Relationship between child exposure IPV, eating-disordered behavior and eating disorders

Three of the 23 studies included in this synthesis examined children’s exposure to IPV, with only one of these sources considering this form of child maltreatment in relation to clinically diagnosed eating-related pathology (Table 4). The strength of the correlation between children’s exposure to IPV and a clinically diagnosed eating disorder was determined to be moderately strong at 0.32 [52]. Importantly, the single self-report measure of eating disorder diagnosis used by the authors asked respondents to indicate presence of a long-term health condition diagnosed by a health professional that had lasted or was expected to last 6 months or longer, a measure that collated all types of eating disorder diagnoses into one item. The unadjusted association between our variables of interest was not reported, thus the correlation recorded here is that which is computed for the most parsimonious model reported by the authors. Two studies [31, 42] considered the relationship between children’s exposure to IPV and eating-disordered behavior, with the correlation between these experiences ranging from very weak (0.04; [31]) to weak-to-moderate (0.21; [31]). Of the two latter studies, one focused on generalized eating-disordered behavior [42] and the other reported correlations between children’s exposure to IPV and participant’s self-reported anorexia and binge eating [31].

### Theoretical frameworks informing eating disorder research among adults with child exposure to IPV, EA, or EN

Only one of the 23 [40] studies (8.7%) identified a theoretical framework informing their research objectives; the work of Burns et al. [40] was informed by the Emotion Regulation Hypothesis [53, 54], which postulates that eating-disordered behavior tempers one’s probability of experiencing negative emotions (e.g. anger, sadness, etc.). Burns et al. [40] argued that child EA could be linked to the experience of eating-disordered behavior through its impact on an individual’s ability to label and regulate their emotions, tolerate the experience of distress and therefore engage in healthy adaptations to stressful life events or experiences. Among their all-women, college sample (*n* = 1254), Burns et al. [40] found that deficits in emotion regulation partially mediated the association between childhood EA and adult eating-disordered behavior.

### Measurement of child maltreatment, eating disorders and eating-disordered behavior

The measures used to assess our child maltreatment variables among the included studies are listed in Table 5. The *Childhood Trauma Questionnaire* [55–57], which is a retrospective self-report tool of child maltreatment history, was the primary data collection measure for 12 of the 23 sources, followed by single-item and author-derived measures. Only three of the sources reported internal reliability consistency estimates for their child maltreatment measure within their given sample [40, 43, 45].

**Table 5:** Measures of child matreatment employed in synthesized studies

Measure (Original Author)	Number of Studies (n)	Citation of Sources Using this Measure
Childhood Trauma Questionnaire [55–57]	(*n* = 12)	Utzinger et al. [33], Mason et al. [30], Michopoulos et al. [17], Moulton et al. [14], Brooke and Mussap [41], Burns et al. [40], Becker and Grilo [36], Bardone-Cone et al. [49], Messman-Moore and Garrigus [43], Fosse and Holen [45], Grilo and Masheb [37], Grilo and Masheb [38]
Childhood Trauma Interview [87]	(n = 1)	Wonderlich et al. [44]
Child Abuse and Trauma Scale [88]	(*n* = 1)	Kent et al. [47]
Childhood Experiences of Violence Questionnaire [89]	(n = 1)	Afifi et al. [52]
Parental Bonding Instrument [90]	(n = 1)	Mullen et al. [51]
Psychological Maltreatment Inventory [91]	(n = 1)	Witkiewitz and Dodge-Reyome [46]
PSY Scale [92]	(n = 1)	Rorty et al. [50]
Trauma Antecedents Questionnaire [93]	(n = 1)	van der Kolk et al. [31]
Author-Specific/Single Item Measures (e.g. “As a child, do you remember being verbally abused?” “While growing up, did you see or hear family violence-such as your gather hitting your mother, or any family member beating up or inflicting bruises, burns or cuts on another family member?”)	(n = 3)	Feinson and Hornik-Lurie [32], Gentile et al. [42], Kugu et al. [39]

Measures used to assess eating disorders and eating-disordered behaviors are summarized in Table 6. Among the sources focusing on clinically diagnosed eating disorders, the *Structured Clinical Interview for DSM – IV Axis I Disorders* [58, 59] was the primary method for diagnostic assessment in five [33, 37–39, 49] of the nine studies. The *Eating Disorder Examination Questionnaire* [60, 61] was the primary data collection measure for studies examining eating-disordered behavior, followed by the *Eating Disorder Inventory (EDI)* [62–64] and author-derived measures. One source [41] cited more than one measure to assess various aspects of eating-disordered behavior and one additional source [38] utilized a diagnostic interview as well as self-report measures in their work.

**Table 6:** Measures of eating disorders/eating-disordered behavior employed in synthesized studies

Measure (Original Author)	Number of Studies (n)	Citation of Sources Using this Measure
Self-Report Measures		
Eating Disorder Examination [60, 61]	(*n* = 5)	Moulton et al. (2015), Burns et al. (2012), Becker and Grilo (2011), Wonderlich et al. (2007), Grilo and Masheb (2001)
Eating Disorder Inventory [62, 64]	(*n* = 4)	Brooke and Mussap (2013), Messman-Moore and Garrigus (2007), Witkiewitz and Dodge-Reyome (2000), Kent et al. (1999),
Drive for Thinness Subscale of the Eating Disorder Inventory – 3 [63]	(n = 1)	Brooke and Mussap [41]
Drive for Muscularity Scale [94]	(n = 1)	Brooke & Mussap [41]
Dutch Eating Behavior Questionnaire [95]	(n = 1)	Michopoulos et al. [17],
Eating Disorder Diagnostic Scale [96, 97]	(n = 1)	Gentile et al. [42]
Adapted Impulse-Anger Checklist [98]	(n = 1)	van der Kolk et al. [31]
Author Specific and/or Single Self-Report Item	(n = 4)	Feinson and Hornik-Lurie [32], Mason et al. [30], Afifi et al. [52], Fosse and Holen [45]
Clinical Interview		
Structured Clinical Interview for DSM-IV Axis I Disorders [58, 59]	(n = 5)	Utzinger et al. [33], Bardone-Cone et al. [49], Kugu et al. [39], Grilo and Masheb [37], Grilo and Masheb [38]
The Composite International Diagnostic Interview (CIDI) [99]	(n = 1)	Schoemaker et al. [34]
Eating Disorder Version of the Schedule for Affective Disorders and Schizophrenia (EAT-SADS-L) [100]	(n = 1)	Rorty et al. [50]
Present State Examination (PSE) – Short Form [101, 102]	(n = 1)	Mullen et al. [51]

### Mediators, moderators and the consideration of socio-economic status

#### Mediators

Five sources evaluated potential mediators between child maltreatment and eating-related pathology, postulating mechanisms by which EA, EN and child exposure to IPV are related to eating disorders and eating-disordered behavior in adulthood. Three sources investigated the extent to which deficits in emotion regulation mediated the relationship between child EA and global eating-disordered behavior in adulthood, with all three of the sources indicating that emotion regulation deficits partially [40] or fully [14, 17] mediated the relationship between these experiences. Two sources found that dissociative symptoms fully mediated the relationship between these child maltreatment experiences and self-reported eating-disordered behaviors in adulthood [14, 47]. In the work by Feinson and Hornik-Lurie [32], the authors found that anger and self-criticism fully mediated the association between child EA and binging behavior among a cross-sectional sample of women (≥ 21 years). Depressive and anxious symptoms were not significant mediators in the model including both self-criticism and anger. Contrary to this, depression and anxiety were found to mediate the relationship between child EA and global, self-reported assessments of eating-disordered behavior among other cross-sectional, women-dominant, community [17] and college-based [47] samples.

#### Moderators

Three of the 23 sources considered moderators of the association between our child maltreatment variables of interest and eating-disordered behavior in adulthood. These moderators included: age of child maltreatment onset, gender and race. With respect to age of child maltreatment onset, results from Kent and colleagues’ [47] study involving a community-based sample of 236 women showed that this maltreatment characteristic did not moderate the mediational association between EA, anxiety and eating pathology, nor the mediational association between EA, dissociation and eating-disordered behavior. Similarly, Brooke and Mussap [41] hypothesized that drive for thinness would be associated with childhood maltreatment among women only. However, results of the hierarchical regression analysis with their cross-sectional college-age sample found no significant interaction between gender, EA or EN in the association with drive for thinness, thereby precluding the ability to assert that compared to men, women who experience EA or EN experience a greater drive for thinness. Similarly, Gentile and colleagues’ [42] cross-sectional survey of college students in the US set out to determine the extent to which participant gender and race modified the association between child exposure to IPV and eating-disordered behavior in adulthood. Given that a main effect for this child maltreatment variable on eating-disordered behavior was not found, the interaction analyses were not completed.

#### Socio-economic disadvantage

Only six of the 23 studies considered participant socio-economic disadvantage or a proxy of this experience in their analyses, with the metrics of this assessment varying considerably. All but one of the sources [30] incorporated multiple indicators of participants' disadvantage in their respective analyses. The specific indicators of disadvantage across these sources included: social status [30], annual household income [52], monthly household income [17], highest level of education completed by the participant [17, 51, 52], full versus part-time student status [44], employment status [17], receipt of disability benefits [17], receipt of financial aid at school [42], annual household income of less than $50,000 (US) [42], current occupation [51] and change in socioeconomic disadvantage from childhood to adulthood [51]. All six of the sources controlled for disadvantage (or its proxy) in multivariate analyses, but did not provide the empirical estimate generated for this variable in their results, nor did any of the papers consider disadvantage from an explanatory perspective in their analytical framework.

### Considerations of maltreatment co-occurrence

Eleven of the 23 sources included in this synthesis controlled for other forms of child maltreatment in their analyses [14, 31, 34, 40, 41, 43–45, 47, 49, 52]. Among these eleven sources, all controlled for physical abuse and sexual abuse, four sources additionally controlled for physical neglect [14, 41, 44, 49] and two additionally considered a combined emotional and physical neglect variable in their analyses [31, 47]. Notably, none of the sources which focused on the association between EA and EN controlled for childhood exposure to IPV. Three of the studies reported standard descriptive statistics for our study-related child maltreatment variables, but then combined the child maltreatment variables of interest with other forms of child maltreatment in regression analyses (e.g. by using a total child maltreatment score) [17, 33, 42], precluding the ability to discern the influence of specific forms of child maltreatment on eating-disorder outcomes. Six of the included sources examined the correlation or association between other forms of child maltreatment (e.g. physical abuse, sexual abuse or physical neglect) and eating-related concerns, but did so without adjusting or controlling for the potential co-occurrence of child EA, EN or exposure to IPV [30, 38, 39, 46, 50, 51]. That is, they looked at the unadjusted, independent association between various forms of child maltreatment without controlling for other types of child maltreatment in their analyses. Finally, two of the included sources in this synthesis did not consider any additional form of child maltreatment (i.e. physical or sexual abuse, or physical neglect) [32, 37], with one study being unclear with respect to whether or not it controlled for other forms of maltreatment [36].

## Discussion

The primary objective of this review was to systematically search and critically synthesize the quantitative literature evaluating the relationship between child EA, EN, and exposure to IPV and eating-related pathology in adulthood. Results reveal a dearth of literature in this area, particularly in relation to the influence of child exposure to IPV on adult eating disorders and eating-disordered behavior. Importantly, the prevalence of EA and EN among individuals with BN, BED and binge eating symptoms appears to be high (21.1% to 66.0%), but the nature and strength of correlation between these forms of child maltreatment and eating-related pathology can be considered inconclusive at best. More specifically, findings from this synthesis indicate that the available evidence has significant methodological weaknesses and precludes the ability to determine whether these forms of child maltreatment are specific versus non-specific risk factors in the etiology of adult eating disorders, eating-disordered behavior and their variations. In addition, the available evidence provides a gendered perspective, with 87% of the included sources having female-dominant samples, although this gendered focus fits with the disproportionate rate of eating and weight-related concerns experienced by the female population [23]. Studies were inconsistent with respect to reporting the proportion of sample participants who identified as an ethnic minority or as living in an immigrant family. This is particularly concerning given that these are two demographic characteristics whereby compared to their non-immigrant and White peers, conflicting information in the antecedents, correlates, prevalence, interventions and outcomes pertaining to child maltreatment [65–67] and eating disorders [68–72] has been found. Less than a handful of the studies examined child EA, EN, and exposure to IPV in relation to AN, ARFID, OS-FED, or the behaviors of purging, excessive exercise, laxative, diuretic or steroid use or abuse. These are significant gaps in the literature.

Only one of the included studies [40] situated their work within a theoretical framework [i.e. emotion regulation hypothesis; 53, 54], postulating that the development of eating disorders and eating-disordered behavior can be considered a maladaptive coping strategy in response to the experience of EA. The absence of theory in the synthesized literature is particularly compelling given that a number of sources postulated potential mediators (e.g. depressive symptoms) and moderators (e.g. gender, age of child maltreatment onset) of the child maltreatment and eating pathology relationship, thereby implicitly suggesting an explanatory pathway by which these phenomenon are related. Importantly, it is the theoretical framework which delineates the proposed explanatory influence of the variables of interest and therefore gives meaning and understanding to the etiology of the relationships found. Thus, it is difficult to postulate the extent to which any one specific model holds greater utility or explanatory influence than another.

It is possible that a theoretical grounding in the transdiagnostic framework for emotional disorders [e.g. see 73] may be useful for future work investigating and understanding the inter-relationships between various forms of child maltreatment; the onset, prevalence and duration of eating disorders and eating-disordered behavior, and other socio-ecological risk and protective factors that have been found to underlie these experiences. As outlined above, child maltreatment is associated with a range of mental health conditions that are often comorbid with eating-related pathology [73], and a few studies in the present review identified potential mediators (such as emotion dysregulation, among others) of the child maltreatment and eating disorder relationship. The transdiagnostic approach takes into account the considerable overlap of various mental health sequelae as well as risk factors (e.g. child maltreatment) for these outcomes, so may be useful in identifying approaches to intervention. Furthermore, increasingly, there is an emphasis on taking an intersectional perspective when investigating health outcomes [74–77], such that multiple aspects of an individual’s identity are considered within the context of micro and macro influences on health and wellbeing. Irrespective of its form, we would advocate that theory must remain the crux of research endeavours, as it is the platform from which questions of clinical and practical salience are justified and empirically evaluated.

Of note, across this synthesis, assessment of child EA, EN or exposure to IPV did not, in any study, evaluate the extent to which the child maltreatment exposure was characterized by ridicule, degradation, humiliation, shame or neglect in relation to the respondents’ body weight, shape or appearance. Nor did any evaluate whether participants were exposed to these forms of degradation between their caregivers. Previous research has found that exposure to family-based teasing about appearance, weight or shape in childhood or adolescence is associated with eating-disorder pathology in adulthood [78–80]. It is possible that abusive remarks or behaviors that centre on weight and/or shape are more strongly associated with eating pathology compared to other types – for example, about intellectual or physical disability, among others.

Another limitation identified within the included studies was the lack of attention to the duration of exposure to abuse in childhood more broadly. Measurement of maltreatment generally referred to the broad time period prior to 18 years of age. It is therefore largely unclear the extent to which child maltreatment during different developmental time periods accounts for the onset, variability, severity and duration of eating-disordered behaviors and eating disorders over the life course. For example, low-severity, chronic maltreatment starting in early childhood could have very different effects compared to a severe, singular experience. It is prudent to consider not only different types of childhood maltreatment in future research, but the chronicity and severity of these experiences as well.

The most appropriate strategy for assessing and identifying child maltreatment and eating-disordered experiences is still under debate. Boyle and colleagues [81] suggest that the use of self-reported questionnaires or checklists can lead to certain benefits compared with the use of semi-structured or structured diagnostic interviews. These benefits include the ability to capture greater variability in the experience or symptoms of the outcome of interest, the ability to dilute potential bias that may be attributable to participant-interviewer interaction, the ability to reduce response burden on behalf of participants and the potential for greater yield in sensitive information that may be more amenable to non-verbal solicitation [81]. There is also the potential for significant cost savings in the use of self-report questionnaires given that structured clinical interviews are typically time consuming and too costly for community-based longitudinal investigations. Unfortunately, the present synthesis precludes our ability to recommend any one specific measure of child EA, EN, or exposure to IPV, or a specific form of assessment. Rather, our goal was to provide descriptive information regarding the measures utilized, as well as to note important considerations for moving the field forward. Of relevance for future epidemiological and clinical research is that only a handful of the included studies provided validity and reliability estimates for their given assessment procedure (i.e. self-report or interview) within their study sample and none of the included studies employing self-report questionnaires evaluated the equivalence of their study measure prior to making cross-group comparisons on their child maltreatment or eating disorder variable.

Adequately powered, representative studies capable of measuring and evaluating the independent and intersecting experiences of various forms of child maltreatment on eating-related pathology are needed. Unfortunately, none of the included sources considered all forms of child maltreatment in their analyses nor did they consider the caregiver characteristics of participants’ child maltreatment experiences. These omissions preclude our ability to make any conclusions in relation to the most salient form of child maltreatment implicated in the etiology of eating disorders; they also do not allow us to make any conclusions about caregiver characteristics.

The inconsistent and limited consideration of the role of socioeconomic disadvantage in the reviewed literature further complicates an already unclear understanding about the influence of this variable in independent and intersecting experiences of child maltreatment and eating disorders. While indicators of disadvantage tend to be associated with child EA, EN and exposure to IPV [82, 83], literature also shows that socioeconomic disadvantage tends to be associated with significant psychiatric morbidity and mortality, with eating disorders potentially being an exception to this norm. A review by Mitchison and Hay [84] reports inconsistent findings between socioeconomic disadvantage and eating disorders; the authors suggest that its indicators do not appear to have strong associations with eating disorders. Importantly, however, one must consider that few adults with eating disorders seek or receive appropriate treatment for their eating-related concerns [85] and that generally, individuals with psychiatric conditions and socioeconomic disadvantage experience disproportionately lower access to mental health services [86]. Thus it is likely that individuals with socioeconomic disadvantage who have a history of child maltreatment and who are living with eating-disordered pathology are at even greater risk for long-term morbidity and mortality compared to their non-socioeconomically disadvantaged, non-maltreated, peers.

### Strengths and limitations

The strengths of this review include the use of systematic searching and citation chaining to identify sources for the synthesis, the use of clear a-priori inclusion and exclusion criteria, and the quality appraisal of studies using an established appraisal system. Our review incorporated search terms and strategies that reflect enhanced understanding about the subtypes of both child maltreatment and eating disorders, however, there is still considerable variability in the use of these terms.

This review focused on English-language studies using quantitative methods and which evaluated the correlation or association between EA, EN, child exposure to IPV and eating disorders and eating-disordered behavior among adults. As such, it does not provide critical commentary on the quality or nature of the relationship between child maltreatment and eating disorders as captured in the qualitative literature. In addition, our review does not comment on the relationship of these experiences among adolescents—a population for whom eating disorders present as a significant concern. Therefore, a similar review amongst this population that focuses on the nature of these experiences (i.e. qualitative perspectives) would complement the findings contained in this review. Finally, our review does not evaluate factors which are predictive of resilience following experiences of child maltreatment or factors which may protect maltreated individuals from developing eating-related concerns.

## Conclusion

As independent and intersecting public health concerns, child maltreatment and eating disorders are associated with significant morbidity, mortality and economic burden. The present systematic search and critical review raises important questions about the nature and extent of the literature investigating the relationship between child EA, EN, exposure to IPV and eating-disordered pathology in adulthood. Based on our review, it is clear that a significant proportion of adults with eating-related concerns – namely BN, BED and purging behavior – report a history of these forms of maltreatment in their childhood, however, methodological biases and gaps in the evidence base preclude making any firm conclusion about the nature and strength of the relationships. Our findings indicate that investigations have focused on women, have tended to ignore the experiences of ethnic-minority and immigrant populations, have not examined the variability inherent in eating-disorder pathology and, generally speaking, demonstrate a significant lack of grounding in theory. The latter of these concerns is of particular note, given the consistent—and potentially erroneous—claim by authors that these forms of child maltreatment can be considered non-specific risk factors in eating disorder etiology.

## Additional file

Additional File 1: Medline Search Strategy. This is a sample search strategy from our systematic literature search. This search strategy was used to identify and extract relevant records from the Ovid (Medline) database. (DOCX 14 kb)

## Abbreviations

AN: Anorexia Nervosa
ARFID: Avoidant Restrictive Food Intake Disorder
BED: Binge Eating Disorder
BN: Bulimia Nervosa
CM: Child Maltreatment
EA: Emotional Abuse
ED: Eating Disorder
EDB: Eating Disordered Behavior
ED-NOS: Eating Disorder Not Otherwise Specified
EN: Emotional Neglect
IPV: Intimate Partner Violence
OS-FED: Other-Specified Food or Eating Disorder
QUIPS Tool: Quality in Prognosis Studies Tool
