# Supplementary material for: Consequences of child emotional abuse, emotional neglect and exposure to intimate partner violence for eating disorders: a systematic critical review
Source: BMC Psychol. 2017 Sep 22;5:33. doi: 10.1186/s40359-017-0202-3 (PMC5610419; doi:10.1186/s40359-017-0202-3)
Supplement: Additional File 1: — Medline Search Strategy. This is a sample search strategy from our systematic literature search. This search strategy was used to identify and extract relevant records from the Ovid (Medline) database. (DOCX 14 kb) [file 40359_2017_202_MOESM1_ESM.docx]

Supplementary File 1

Medline Search Strategy

Database: Ovid MEDLINE(R) In-Process & Other Non-Indexed Citations, Ovid MEDLINE(R) Daily and Ovid MEDLINE(R) <1946 to Present>
Search Strategy:
--------------------------------------------------------------------------------
1     mental disorders/ or eating disorders/ or anorexia nervosa/ or binge-eating disorder/ or bulimia nervosa/ or female athlete triad syndrome/ (150516)
2     (mental adj5 (disorder* or psychopathol* or patholog* or problem? or illness*)).ti,ab. (61728)
3     mental health.ti,ab. (88591)
4     (eating adj5 (disorder* or psychopathol* or patholog*)).ti,ab. (15232)
5     (bulimia* or bulimic* or anorexia or "eating disorder not otherwise specified" or EDNOS).ti,ab. (27834)
6     ((binge or binging or purge or purging or vomit*) adj5 (food? or eat or eating or diet? or dieting)).ti,ab. (4765)
7     (athlete* adj5 triad).ti,ab. (223)
8     (emotion* adj5 (food? or eat or eating or diet? or dieting)).ti,ab. (1482)
9     (extreme adj5 (diet? or dieting)).ti,ab. (176)
10     (calor* adj5 restrict*).ti,ab. (6176)
11     ((diet? or dieting or weight or laxative? or purgative? or aperient?) adj5 (pill? or medication? or drug? or prescription?)).ti,ab. (10037)
12     or/1-11 (277486)
13     Child Abuse/ (18607)
14     ((child* or girl? or boy? or toddler? or preschool* or pre-school* or pre school* or youth* or (young* adj2 (person? or people? or individual?)) or minor? or teen* or adolescen* or preteen* or tween* or kid? or son or sons or daughter? or grandchild* or grandson? or granddaughter? or offspring?) adj5 (abuse? or abusing or maltreat* or neglect*)).ti,ab. (23498)
15     or/13-14 (31801)
16     spouse abuse/ or battered women/ or domestic violence/ (12063)
17     ((spous* or partner?? or wife or wives or husband? or family or families or domestic* or intimate* or conjugal* or marital* or interparent* or interpartner*) adj3 (abus* or violen* or batter or battered or batters or batterer? or battering)).ti,ab. (12512)
18     or/16-17 (16728)
19     adolescent/ not (adolescent/ and exp child/) (927562)
20     exp child/ or adolescent/ (2565964)
21     (child* or girl? or boy? or toddler? or preschool* or pre-school* or pre school* or youth* or (young* adj2 (person? or people? or individual?)) or minor? or teen* or adolescen* or preteen* or tween* or kid? or son or sons or daughter? or grandchild* or grandson? or granddaughter? or offspring?).ti,ab. (1577218)
22     (20 not 19) or 21 (2395674)
23     18 and 22 (6723)
24     15 or 23 (35339)
25     12 and 24 (4354)
26     [adult.mp](http://adult.mp). or middle aged.sh. or age:.tw. (7338227)
27     (woman or women or man or men).tw. (1282081)
28     or/26-27 (7701299)
29     25 and 28 (3216)
